# Prediction of long-term remission in patients following discontinuation of anti-TNF therapy in ulcerative colitis: a 10 year follow up study

**DOI:** 10.1186/s12876-022-02522-4

**Published:** 2022-11-16

**Authors:** Kay-Martin Johnsen, Jon Florholmen, Øystein K. Moe, Mona Gundersen, Julia Beilfuss, Hege Kileng, Sveinung W. Sørbye, Rasmus Goll

**Affiliations:** 1 Research Group of Gastroenterology and Nutrition, Department of Clinical Medicine, UiT The Arctic University of Norway, Tromsö, Norway; 2 Division of Internal Medicine, Department of Gastroenterology, University Hospital of North Norway, Tromsö, Norway; 3 Department of Clinical Pathology, University Hospital of North Norway, Tromsö, Norway

**Keywords:** Anti-TNF therapy, Biological therapy, Inflammatory bowel disease, Tumor necrosis factor, Ulcerative colitis

## Abstract

**Background:**

The long-term outcomes of Ulcerative colitis (UC) after discontinuation of biological therapy are largely unknown. There is also a lack of accurate and validated markers that can predict outcome after withdrawal accurately. The aims of this study were to describe the long-term outcomes in UC patients following cessation of anti-TNF therapy and explore potential biomarkers as an approach towards precision medicine.

**Methods:**

Seventy-five patients with moderate to severe UC treated to remission with anti-tumor necrosis factor (TNF) were included in the study. This is a follow-up of previously reported UC outcomes. The patients were categorized as either “Remission” or “Relapse”. The “Relapse” group was divided into subgroups determined by the highest treatment level needed to obtain remission the last 3 years of observation: non-biological therapy, biological therapy or colectomy. Remission were divided in long term remission (LTR), those using immunomodulating drugs (LTR + imids) and those using only 5-amino-salicylate (5-ASA) treatment (LTR) for the past 3 years. Analyses of mucosal gene expression by real-time PCR were performed.

**Results:**

The median (IQR) observation time of all patients included was 121 (111–137) months. Of the 75 patients, 46 (61%) did not receive biological therapy, including 23 (31%) in LTR ± imids. Of these 23 patients, 16 (21%) were defined as LTR with a median observation time of (IQR) 95 (77–113) months. In total 14 patients (19%) underwent colectomy during the 10 years after first remission. Mucosal TNF copies/µg mRNA < 10 000 at anti-TNF discontinuation predicted long-term remission, biological free remission and lower risk of colectomy with a HR 0.36 (0.14–0.92) for long-term remission, HR 0.17 (0.04–0.78) for biological free remission and HR 0.12 (0.01–0.91) for colectomy. IL1RL1 was normalized in LTR phenotype and higher in relapsing UC.

**Conclusion:**

In this 10-year follow-up of UC of patients with moderate to severe disease, 61% of patients experience an altered phenotype to a milder disease course without need of biological therapy. Twenty-one percent of the patients were LTR without any medication except of 5-ASA. Mucosal TNF gene expression and IL1RL1- transcripts may be of clinical utility for long term prognosis in development of precision medicine in UC.

**Supplementary Information:**

The online version contains supplementary material available at 10.1186/s12876-022-02522-4.

## Introduction

Ulcerative colitis (UC) is regarded as a lifelong chronic inflammatory disease most likely caused by genetic and environmental factors where the disease course on an individual level varies greatly [1–4]. One of the most referred studies for clinical outcomes and disease course of UC is from the IBSEN group [5]. During a 5-year period after diagnosis, 59% reported a decline in self-reported symptom burden, whereas 1%, 9% and 31% reported increasing activity, chronic continuous and, chronic relapsing disease, respectively. In a 10 year follow-up study of UC patients, 55% reported a disease course in remission or only mild symptoms [6].

In a recent review of population-based cohorts mainly including mild and moderate adult UC, 10–15% experienced an aggressive disease course with a 10 years cumulative risk of relapse as high as 70–80%, and a colectomy rate of 15% [7].

Biological therapies for UC, including anti-tumor necrosis factor (anti-TNF) have shown high efficacy in achieving disease remission- after 12 months [8–10]. However, loss of response in UC on biological maintenance therapy have been reported to be in the range of 15 to 20% annually [11]. Moreover, the one-year risk of relapse after ani-TNF withdrawal in UC can be as high as 50% and as low as 6% [12]. There is a lack of reports regarding long-term outcomes after discontinuation.

So far there are no risk factors, neither clinical, immunological, genetic or laboratory markers that can predict outcome after withdrawal accurately [13, 14]. Therefore, neither published AGA or ECCO guidelines include clear recommendations regarding withdrawal of biological therapy [13–15].

We have previously published results showing that normalization (< 10 000 copies/µg mRNA) of mucosal TNF mRNA when discontinuing anti-TNF treatment in both UC and Crohn´s disease prolonged the time in remission. In UC the median time to relapse was 36 months, compared to 11 months in the group with increased mucosal TNF mRNA level [16, 17]. These reports are of great value in the effort of obtaining predictive biomarkers suitable for a precision medicine algorithm with individualized therapy in UC [18–23].

This is a follow-up of the previously reported prospective UC outcome study [16] with 4 years extended outcome registrations until 2019 with the following aims:

1. To define the immunological phenotype (fingerprint) of long-term remission.
2. Define a subgroup of patients who are in long-term remission with only 5-ASA per oral maintenance treatment or no medical treatment (LTR).
3. Describe potential biomarkers to predict long-term remission following discontinuation of anti-TNF therapy; i.e. an approach to development of precision medicine.

## Materials and methods

**Fig. 1:**
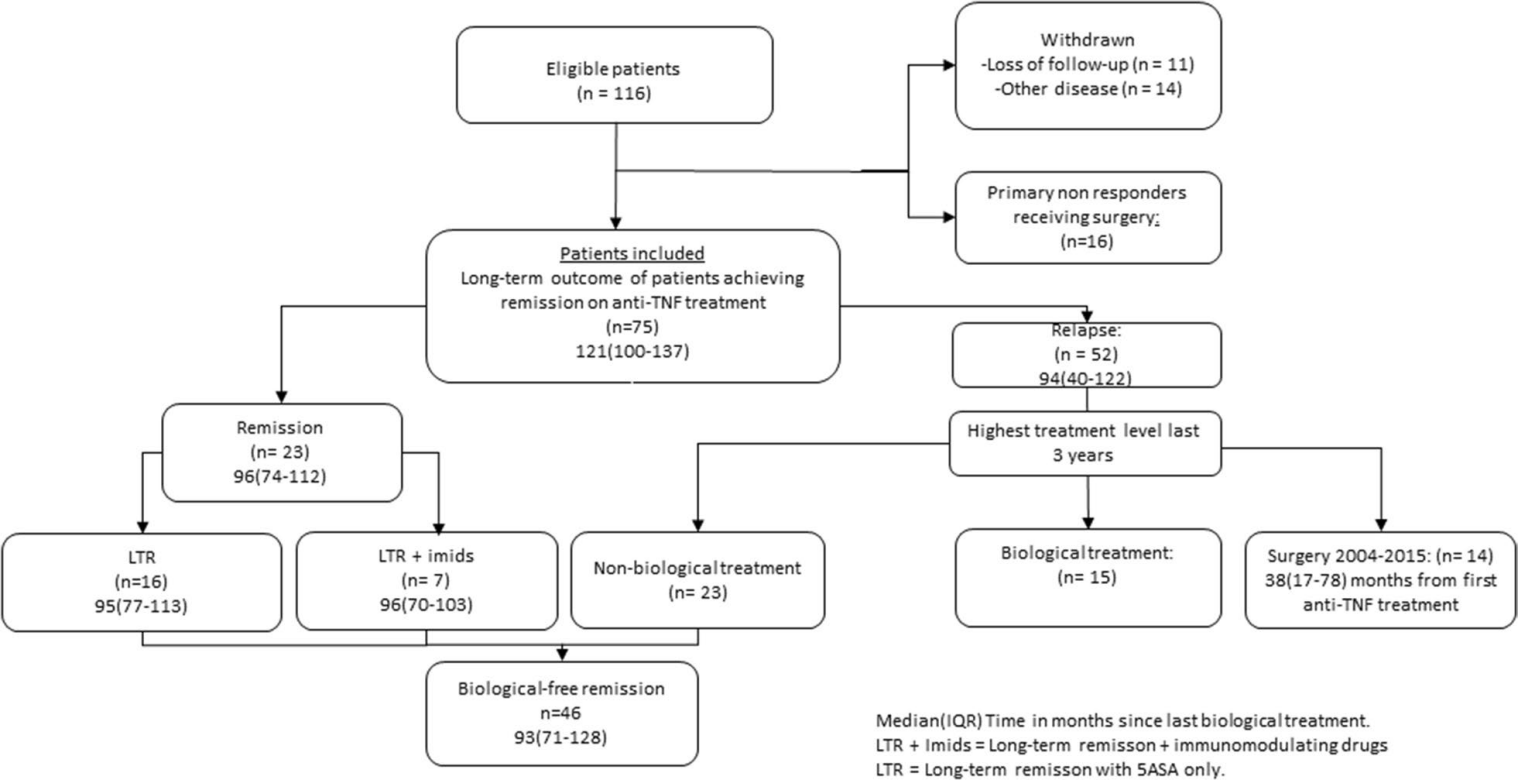
Flow chart of long-term outcomes of patients treated with anti-TNF treatment

This study is part of the ongoing prospective transregional study Advanced Study of Inflammatory Bowel disease (ASIB- study). All patients included in this follow-up were previously treated to remission with anti-TNF, subsequent discontinuation, and retreatment in case of relapse. In the first report of clinical outcomes in UC patients following this algorithm, 116 patients were included. Of these 116 patients, 96 patients obtained remission and observed until August 2015 [16]. The patients included and follow-up are described in Fig. 1. In addition, 24 healthy controls (HC) and 9 patients with UC that relapses within 12 months after discontinuation of IFX were also recruited (early relapse group). The HC and the early relapse, were included to measure mucosal gene transcripts to compare with patients from the follow up with > 3 years in remission to find potential predictors of remission.

### Categorization of long-term outcome after stop of anti-TNF

Patients were divided into predefined groups termed “Relapse” or “Remission”. The “Relapse” group was further divided into subgroups determined by the highest treatment level needed to obtain remission in the last 3 years; Biological therapy, colectomy or non-biological therapy including; corticosteroids, immunomodulators, 5-ASA. Corticosteroid use was short term courses when needed. No patients received integrin blockers or JAK inhibitors. Patients in the Remission group, had no relapse during the study period following discontinuation of the last anti TNF treatment. The remission group was divided into two subgroups, long-term remission without any treatment other than 5-ASA (LTR) and with immunomodulating drugs (LTR + imids). The definition of LTR was minimum 3 years in clinical remission after stopping/discontinuing anti-TNF. Clinical remission was defined by a combination of global assessment using ulcerative colitis Clinical Score (UCCS) less than 3 and faecal calprotectin < 100 mg/kg. Patients in LTR were invited to a follow-up endoscopy and clinical evaluation with UCDAI score.

### Criteria for discontinuation of anti-TNF treatment

The criteria of discontinuation of anti-TNF treatment were endoscopic remission with a Mayo endoscopic sub-score of 0–1, until these 4 years extended observation time whereas an additional criterium of clinical remission > 6 months and normalized mucosal TNF gene expression were included in 2014 [24, 25].

### Relapse

Relapse was defined as a clinical, biochemical, endoscopic signs of disease activity leading to a therapeutic intervention as escalation of medical therapy or surgery.

### Healthy control group

We included 24 healthy controls, 8 and 16 females and males respectively, with median (25–75 percentile) of 57 (42–67) years. The patients were recruited in the time period March 2014 to March 2018. The healthy control included patients referred for cancer screening where colonoscopy was normal. Exclusion criteria were serious medical conditions including immunological disorders, irritable bowel symptoms, polyps or cancer and abnormal histology in colonic biopsies.

### Tissue samples

We performed tissue sampling by using standard forceps and retrieving two mucosal biopsies in witch we performed all cytokine measurements. Colonic mucosal biopsies were sampled from the region with most severe inflammation in patients with active inflammation. In patients in remission, biopsies were sampled from the previously most inflamed region. Biopsies were sampled from the sigmoid in healthy controls. The biopsies were histologically assessed by an experienced pathologist (SWS) using Robarts histopathology index (RHI) score [26]. The samples for RNA extraction were immediately immersed in RNA *later* (Qiagen), and stored at room temperature overnight, then at – 20 °C until RNA isolation.

### Cytokine measurements

Real-time PCR procedures have previously been described in detail [25, 27, 28]. Total RNA was isolated from mucosal biopsies using the Allprep DNA/RNA Mini Kit (Qiagen, Hilden, Germany, Cat No: 80204) and the automated QIAcube instrument (Qiagen, Hilden, Germany) according to the manufacturer’s recommendations. Quantity and purity of the extracted RNA were determined using the Qubit 3 Fluorometer (Cat No: Q33216; Invitrogen by Thermo Fisher Scientific, Waltham, MA, USA). Reverse transcription of the total RNA was performed using the QuantiTect Reverse Transcription Kit (Cat. No: 205314; Qiagen, Hilden, Germany) and QuantiNova probe RT-PR kit (Quiagen Cat no:208352). Primer sequences are previous published [29, 30]. The following gene transcripts were measured*: IL17a, IL23, IL4, IL5, IL6, IL10, IL21, IL33, IL1B, TGFβ, GATA, IL18, TLR4, IL1RL1, RORC, FOXp3, TBX21* and *TNF*.

### Statistics

Two-way ANCOVA models adjusted for sex, disease distribution and age were performed to compare cytokine levels between groups and estimate effect size. For cytokines with a *P*-value of < 0.05, the difference in ΔΔCT fold change were calculated and converted to fold difference indicating up or down regulated in LTR compared to HC and early relapse group. To evaluate predictors of surgery, biological free remission and remission without relapse, Cox regression analyses were performed and receiver operating characteristics (ROC) curves were constructed. All statistical analyses were carried out in IBM SPSS Statistics 24 (IBM Corporation, Armonk, New York, USA).

## Results

### Clinical groups and demographic characteristic

All patients included, including subgroup division are presented in Fig. 1. Demographic characteristics of patients included and main selected subgroups are shown in Table 1. The median observation time of all patients included was 121 (111–137 IQR) months.

**Table 1:** Demographic data of patients after stop of anti-TNF

Patient groups	Included N = 75	Remission N = 23	LTR N = 16	Relapse N = 52	Early Relapse N = 9
Age med (IQR)	46 (38–55)	51 (39–56)	41 (26–55)	46 (37–54)	46 (29–56)
Sex Female/Male	29/46	9/14	8/8	20/32	4/5
Colonic area R/L/E	10/30/35	4/9/10	1/9/6	6/21/25	0/5/4
UCDAI score med (Range)	0 (0–4)	0 (0–1)	0 (0–1)	0 (0–4)	0 (0–1)
Observation time (months) med (IQR)	121 (100–137)	96 (74–112)	95 (77–113)	94 (40–122)	4 (4–6)
Medication					
IFX	15	0	0	15	0
5-ASA	55	19	13	23	9
AZA/MTX	30	7	0	23	4
Steroids	4	0	0	4	0

*med (IQR)* median (interquartile range 25–75), *R/L/E* rectum/Left/Extensive, *AZA* azathioprine, *MTX* methotrexate, *LTR* Long-term remission

### Long-term remission

Of the 75 patients included in the study 23 (31%) were still in remission without any clinical, or endoscopic signs of relapse after stop of biological therapy (Fig. 1). The median observation time was 96 (74–112 IQR) months. In the previously described subgroup LTR, 16 patients did not receive any medication except per oral 5ASA (Table 1).

### Relapse

Of the 75 patients, 52 patients relapsed after the last anti-TNF treatment. Fourteen patients received surgery. Fifteen patients were still in need of biological treatment. In the relapse patient group, twenty-three patients obtained remission on non-biological treatment, and of these, 14 patients received immunosuppressive treatment.

### Biological free remission

Forty-six (61%) of the 75 patients included were in biological free remission. This group consists of 23 patients from LTR and 23 patients the “Relapse group” (Fig. 1). The median (IQR) time since discontinuation of biological therapy was 93 (71–128) months.

### Surgery free survival

Of the total 116 patients included in the original study, 30 patients underwent colectomy with a median (IQR) time to colectomy of 18 (8–44) months. From the selected group of 75 patients included in the study that achieved remission on anti-TNF treatment, 14 underwent colectomy during the observation period with a median (IQR) time to colectomy of 38 (17–78) months.

### Mucosal transcripts in ulcerative colitis phenotypes and healthy controls

In Table 2 we show the mucosal gene expression in colonic biopsies sampled in remission before discontinuation of anti-TNF treatment in the two subgroups LTR, and “Early relapse” (ER). Patients in the ER group relapsed within 12 months after anti-TNF discontinuation. Arrows in Table 2 indicates whether the mucosal gene expression in the LTR group is up or downregulated compared to ER and HC respectively. IL1RL boxplot as Additional file 1: Fig. S1.

**Table 2:** Results of cytokine measurements comparing LTR to HC and patients in remission with relapse during the first year after anti-TNF discontinuation

Cytokine	LTR vs. HC		LTR vs. Early relapse	
	*P* value	ΔΔCT FC	*P* value	ΔΔCT FC
IL17	0.026	3.31↑	0.044	5.14↓
IL23	0.000	10.20↑	0.024	2.17↓
IL4	0.024	2.77↑	0.890	ns
IL5	0.329	ns	0.997	ns
IL6	0.001	7.46↑	0.192	ns
IL10	0.000	6.82↑	0.038	2.45↓
IL21	0.062	ns	0.264	ns
IL33	0.001	3.77↑	0.011	2.42↓
TGF	0.007	2.39↑	0.009	1.70↓
GATA	0.000	12.47↑	0.212	ns
TLR4	0.001	3.89↑	0.023	1.69↓
IL1RL1	0.111	ns	0.049	1.93↓
RORC	0.000	6.41↑	0.089	ns
FOX	0.000	8.88↑	0.470	ns
TBX	0.000	5.28↑	0.181	ns
TNF	0.103	ns	0.542	ns

↑ ↓Up or down regulated mucosal gene expression in LTR group

*CT FC* Cycle threshold fold change, *ns* not significant, *LTR* Long-term remission, *HC* Healthy control

### Long-term remission versus healthy control

Patients in LTR were invited to a follow up endoscopy and colonic biopsies were sampled. There were no significant differences between HC and LTR for TNF, IL1RL1, IL5 and IL21 indicating normalization in LTR. However, the other proinflammatory cytokines and T-regs were increased in this group compared to healthy controls, despite being in endoscopic remission.

### Long-term remission versus early relapse

Colonic biopsies were sampled from the early relapse group at time of discontinuation of anti-TNF treatment and histologically assessed. With the exception of one patient in the LTR group, all included colonic biopsies were histologically assessed as RHI 0. The patient in the LTR group were assessed as RHI 4. Significantly increased values in the ER group compared to the LTR group were seen for cytokines such as IL10, 1IL17, IL23, IL33, TLR4 and TGF. Of interest, the IL1RL1 transcript was normalized in the LTR, but increased in the ER group compared to the LTR and HC.

### Prediction of long-term remission and biological free remission

**Fig. 2:**
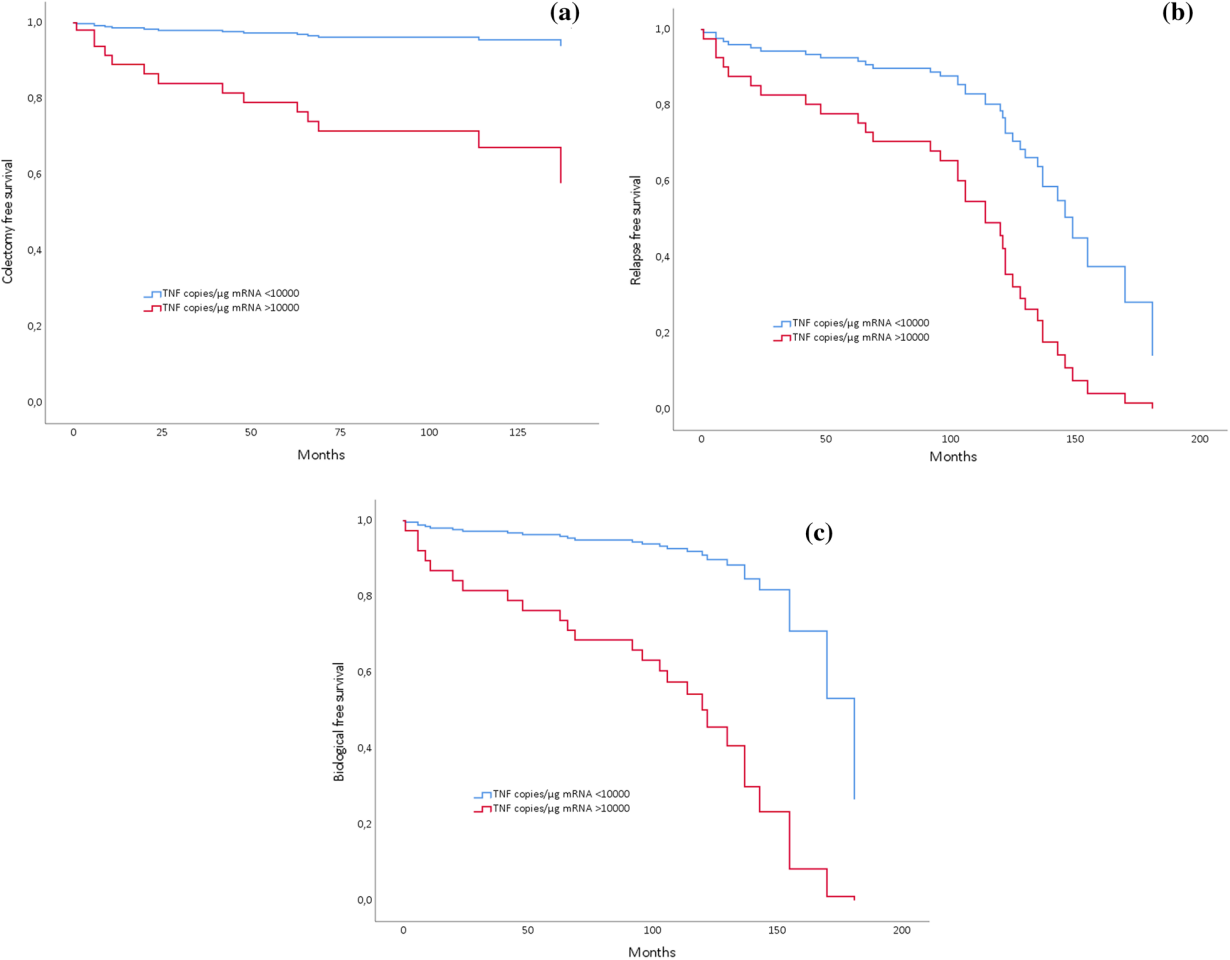
**a**–**c** Cox regression model entering mucosal TNF gene expression normalization after first anti-TNF treatment and age at inclusion. **a** Model *P* = 0.003; low TNF at IFX discontinuation: HR 0.12 (0.01–0.91) for surgery (*P* = 0.04); Age baseline HR 1.06 (1.01–1.12) for surgery (*P* = 0.015), **b** Model *P* = 0.005; low TNF: HR 0.31 (0.13–0.76) for long-term remission (*P* = 0.01); **c** Model *P* = 0.001; low TNF: HR 0.13 (0.013–0.61) for biological free remission (*P* = 0.009). *HR* hazard ratio, *IFX* infliximab, *TNF* tumour necrosis factor

**Fig. 3:**
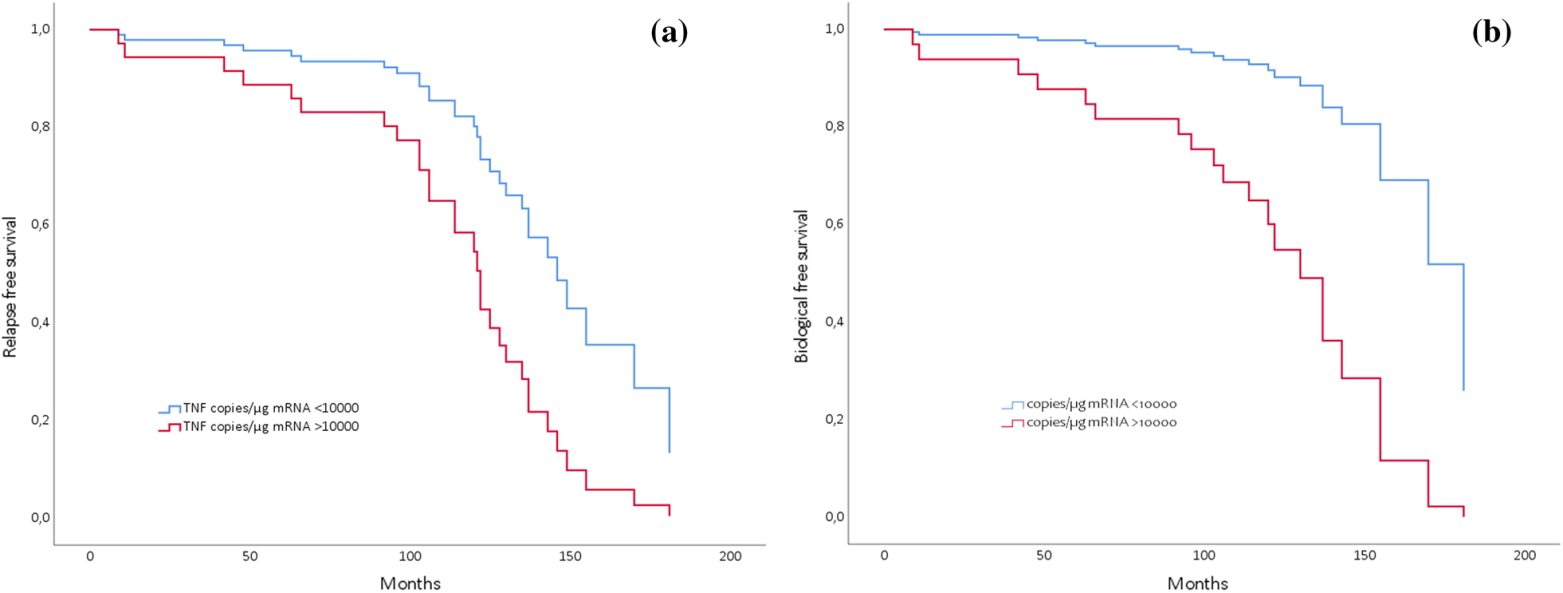
**a, b** Cox regression model entering mucosal TNF gene expression normalization after last anti-TNF treatment. **a** Model *P* = 0.021; low TNF at IFX discontinuation: HR 0.36 (0.14–0.92) for long-term remission (*P* = 0.03). **b** Model *P* = 0.005; low TNF at IFX discontinuation: HR 0.17 (0.04–0.78) for biological free remission (*P* = 0.03). *HR* hazard ratio, *IFX* infliximab, *TNF* tumour necrosis factor

Analysis by Cox regression was carried out including normalized mucosal TNF gene expression (< 10 000 copies/µgRNA), age, disease location, and smoking habits to predict long-term remission and biological free remission. Mucosal TNF gene expression at remission before discontinuation of anti-TNF treatment, after the first and last induction treatment showed significant prediction for both long-term remission and biological free remission (Figs. 2, 3). Of note, 31 and 61 percent of the patients that achieved remission on initial anti-TNF treatment have been in long-term and biological free remission respectively for approximately the last 8 years.

### Prediction of colectomy

Analysis by Cox regression was carried out including normalized mucosal TNF gene expression (< 10,000 copies/µgRNA), age, disease location, and smoking habits to predict colectomy. The resulting model showed a significant effect of normalized mucosal TNF gene expression and young age before the first anti-TNF to predict lower risk of colectomy (Fig. 2). Moreover, ROC analyses were performed by selecting a mucosal TNF gene expression cut off of high 40,000 (copies/µgRNA) at inclusion before start of biological therapy. The test obtained a relative high specificity of 80%, but the sensitivity was as low as 10%. From a clinical point of view this indicates that patients with very elevated mucosal TNF gene expression at debut of disease are in higher risk of colectomy during the disease course.

## Discussion

This 10-year observation study of long-term outcomes after anti-TNF discontinuation upon endoscopic remission, shows that 61% of those who achieve remission do not require biological treatment in the long-term. Fifty percent of the patients without anti-TNF treatment were in clinical remission without any signs of relapse with a median observation time of 8 years, whilst the other half only required escalation of non-biological treatment in case of relapse. This may indicate that anti-TNF treatment may alter the natural disease course for patients with moderate to severe ulcerative colitis.

We have previously described mucosal TNF gene expression as a possible biomarker that predicts prolonged time in remission after anti-TNF discontinuation in ulcerative colitis, and mucosal TNF gene expression combined with histological score predicts need of biological treatment wihtin the first year after diagnosis [18]. In this study we have found that normalized mucosal TNF gene expression at discontinuation of anti-TNF treatment also predicted a milder disease course in the a long-term perspective, and moreover a lower need of biological therapy and lower risk of surgery. A hypothesis could be that patiens with low mucosal TNF gene expression reflects milder disease phenotype. In this context, measuring TNF gene expression could be an analogue to measuring the “temperature” of the local immune activation.

In our former study of 116 patients with moderate to severe UC intention treated with biological therapy (26%) underwent colectomy after a median time of 18 months. Moreover, the selected group of 75 patients included in this study, 19% underwent colectomy after a median time of 40 months during the total observation time of approximately 10 years. In a metanalyses of UC patients, including the majority of the patients with mild to moderate disease, the 10 years cumulative risks of colectomy were 15% [7]. As far as we know there are no clear comparing reports on the risk of colectomy in patients with moderate to severe disease activity, and non-existing with similar observation time after discontinuation biological therapy. Moreover, the need for surgery in patients with moderate to severe UC on maintenance therapy has been reported to be 31% (mean observation time 50 months), 50% (36 months), 27.3% (84 months), 20% (60 months), 53% (60 months) and 17% (33 months), and in a meta-analysis, the mean risk of colectomy after 36 months treatment of IFX in moderate to severe UC was around 40% [31–37]. Normalized mucosal TNF gene expression and young age significantly predicted low risk of surgery. Moreover, at inclusion before start of biological therapy a mucosal TNF gene expression cut off of 40 000 (copies/µgRNA) predicted high risk of surgery with high specificity but low sensitivity. This supports hypothesis of mucosal TNF gene expression reflecting disease severity of UC phenotype. High TNF gene expression levels before start of biological therapy represent patients with high risk of surgery which may be of some clinical utility.

The small subgroups of patients defined as LTR is of special interest according to causal pathophysiological mechanisms of UC. This group was in endoscopic remission with histological healed mucosa. but still not in immunologic remission, i.e. not all mucosal proinflammatory cytokines were normalized. In this LTR group there were no differnce in the mucosal TNF gene expression compared to normal healty controls, but there were still a clear up-regulation of other pro- and anti-inflammatoric mediator genes. This indicates that even in case of long-term endoscopic remission with apparent histological healed mucoa the immune activity was not completely resolved. Moreover, when the LTR patients were compared to other ulcerative colitis patients in remission with normalized mucosal TNF gene expression that experience a relapse within the first year after anti-TNF discontiuation, there is a significant difference in the pro inflammatory pathways including IL17 and IL 23. Apart from a generally lower inflammatory immune acitivety in LTR, the IL1RL1/IL33 pathway is normalized in LTR compared to healthy controls, contradictory to the relapsing phenotype. IL1RL1 is the ligand receptor to the alarmin IL33, and present on a wide range of cells including immune cells in the gut mucosa. Several splice variants of IL1RL1 exist including a membrane receptor (IL1RL1L) as well as a soluble decoy receptor (sIL1RL1). The relationship of mucosal IL-33 and IL1RL1—gene expression is not completly understood with both pro and anti-inflammatory properties described [38]. Final normalisation of IL1RL1 is the most clear fingerprint that differentiate the LTR phenotype from relapsing type, and might be a possible marker of long-term remission without need of treatment escalation. IL1RL1/IL33 activity have in previous studies shown to be a possible marker of inflammatory activiety and corresponds to fecal calprotecin [38]. Our findings support further investigation into the IL1RL1/IL-33 pathway as interest as biomarkers as a step toward precision medicine in UC. This should be adressed in further studies.

The strength of this study is the long-term follow up with systematic registration of oucomes after discontinuation and re-treatment with biological therapy of moderate to severe UC, and the aspect of using features of molecular immunology for development of precision medicine in UC. The weakness of this study is the sample size of the cohort and lack of endoscopical verfication of disease activity. The sample size of the mucosal gene expression analysis is also small. Moreover, an open RCT study design comparing the specific treatment algorithm to manitenance treatment with biological therapy would definately have increased the scientific value. We have only included patients treathed with anti-TNF therapy, and the results cannot necessarily be translated to other biological therapies or smal molecule drugs. This awaits further studies.

In conlusion the study indicates that anti-TNF treatment may alter the disease severity to a milder phenotype for those who do not need colectomy. Low mucosal TNF gene expression in remisson both after the first and folowing induction treatments predict a lower risk of colectomy in the long-term and long-term remission without biological treatment. The mucosal immunologic gene expression profile of LTR is not normalized but shows in generally lower immune acitivety. IL1RL1 is normalized in LTR phenotype and higher in relapsing UC, and normalization of IL1RL1 might be a possible marker of long-term remission.

## Supplementary Information

**Additional file 1: Fig. S1.** Boxplot of IL1RL cytokine measurements comparing HC, LTR and patients in remission with relapse. CT FC= Cycle threshold fold change, HC= Healthy controls, LTR=Long-term remission.

## Data Availability

The raw data are available on shared folder: https://data.mendeley.com/datasets/f4mb2c4nnb/1.

## Abbreviations

ASIB: Advanced study of inflammatory bowel disease
CD: Crohn’s disease
ER: Early relapse
FOXp3: Forkhead box protein3
GATA: Globin transcription factor
HC: Healthy controls
IBD: Inflammatory bowel disease
IFNG: Interferon Gamma
IFX: Infliximab
IL: Interleukin
IL1RL1: Interleukin-1-like-receptor 1
Imids: Immunomodulating drugs
LTR: Long term remission
mRNA: Messenger ribonucleic acid
NFKB: Nuclear factor Kappa B
RORC: Nuclear receptor related orphan receptor C
TBX21: T-box transcription factor 21
TGFβ: Transforming growth factor Beta
TNF: Tumor necrosis factor
UC: Ulcerative colitis
UCDAI: Ulcerative colitis disease activity index
