# Supplementary material for: Prediction of long-term remission in patients following discontinuation of anti-TNF therapy in ulcerative colitis: a 10 year follow up study
Source: BMC Gastroenterol. 2022 Nov 16;22:459. doi: 10.1186/s12876-022-02522-4 (PMC9667633; doi:10.1186/s12876-022-02522-4)

Figure S1 (supplemantory)

Boxplot of IL1RL cytokine measurements comparing HC, LTR and patients in remission with relapse. CT FC= Cycle threshold fold change, HC= Healthy controls, LTR=Long-term remission


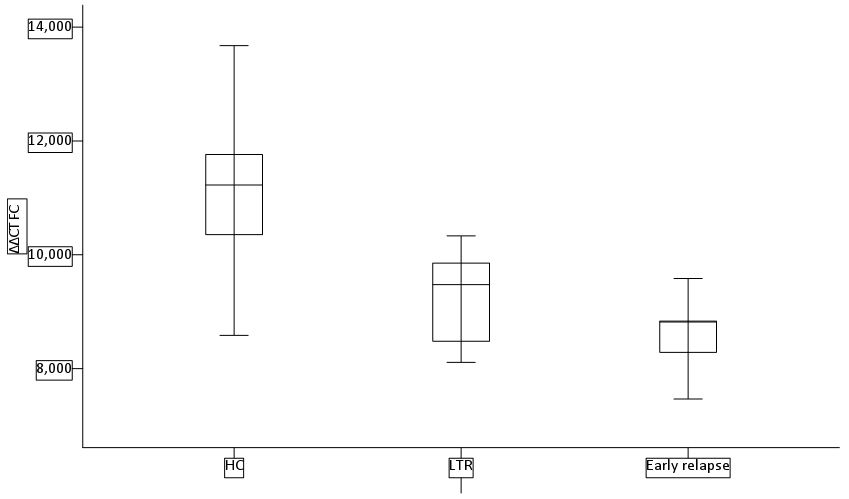

Supplement: Supplementary file 1 — Additional file 1: Fig. S1. Boxplot of IL1RL cytokine measurements comparing HC, LTR and patients in remission with relapse. CT FC= Cycle threshold fold change, HC= Healthy controls, LTR=Long-term remission. [file 12876_2022_2522_MOESM1_ESM.docx]
